# Radiotranscriptomics of non-small cell lung carcinoma for assessing high-level clinical outcomes using a machine learning-derived multi-modal signature

**DOI:** 10.1186/s12938-023-01190-z

**Published:** 2023-12-15

**Authors:** Eleftherios Trivizakis, Nikoletta-Maria Koutroumpa, John Souglakos, Apostolos Karantanas, Michalis Zervakis, Kostas Marias

**Affiliations:** 1 Computational Biomedicine Laboratory (CBML), Foundation for Research and Technology Hellas (FORTH), 70013 Heraklion, Greece; 2 Medical School, University of Crete, 71003 Heraklion, Greece; 3 School of Electrical and Computer Engineering, Technical University of Crete, 73100 Chania, Greece; 4 Laboratory of Translational Oncology, Medical School, University of Crete, 71003 Heraklion, Greece; 5 Department of Medical Oncology, University Hospital of Heraklion, 71500 Heraklion, Greece; 6 Department of Radiology, Medical School, University of Crete, 71003 Heraklion, Greece; 7 Department of Electrical and Computer Engineering, Hellenic Mediterranean University, 71410 Heraklion, Greece

**Keywords:** Multi-omics score, Deep features, Radiomics, Transcriptomics, Integrative data analysis, Non-small cell lung cancer, Survival analysis, Adjuvant chemotherapy response

## Abstract

**Background:**

Multi-omics research has the potential to holistically capture intra-tumor variability, thereby improving therapeutic decisions by incorporating the key principles of precision medicine. The purpose of this study is to identify a robust method of integrating features from different sources, such as imaging, transcriptomics, and clinical data, to predict the survival and therapy response of non-small cell lung cancer patients.

**Methods:**

2996 radiomics, 5268 transcriptomics, and 8 clinical features were extracted from the NSCLC Radiogenomics dataset. Radiomics and deep features were calculated based on the volume of interest in pre-treatment, routine CT examinations, and then combined with RNA-seq and clinical data. Several machine learning classifiers were used to perform survival analysis and assess the patient’s response to adjuvant chemotherapy. The proposed analysis was evaluated on an unseen testing set in a k-fold cross-validation scheme. Score- and concatenation-based multi-omics were used as feature integration techniques.

**Results:**

Six radiomics (elongation, cluster shade, entropy, variance, gray-level non-uniformity, and maximal correlation coefficient), six deep features (NasNet-based activations), and three transcriptomics (OTUD3, SUCGL2, and RQCD1) were found to be significant for therapy response. The examined score-based multi-omic improved the AUC up to 0.10 on the unseen testing set (0.74 ± 0.06) and the balance between sensitivity and specificity for predicting therapy response for 106 patients, resulting in less biased models and improving upon the either highly sensitive or highly specific single-source models. Six radiomics (kurtosis, GLRLM- and GLSZM-based non-uniformity from images with no filtering, biorthogonal, and daubechies wavelets), seven deep features (ResNet-based activations), and seven transcriptomics (ELP3, ZZZ3, PGRMC2, TRAK1, ATIC, USP7, and PNPLA2) were found to be significant for the survival analysis. Accordingly, the survival analysis for 115 patients was also enhanced up to 0.20 by the proposed score-based multi-omics in terms of the C-index (0.79 ± 0.03).

**Conclusions:**

Compared to single-source models, multi-omics integration has the potential to improve prediction performance, increase model stability, and reduce bias for both treatment response and survival analysis.

## Introduction

Despite major advances in the staging, management, molecular profiling, and treatment of non-small cell lung cancer (NSCLC), the disease remains the major cause of cancer deaths worldwide, according to recent reports from GLOBOCAN [1] and the WHO [2]. Genomic profiling of different cancer types could potentially facilitate the identification of new and discriminative biomarkers, allowing for the selection of a treatment plan that is both effective and personalized for the patient [3]. Collecting RNA-seq data is challenging due to the variations in the computational process of this type of data [4], intra-tumor heterogeneity [5], and local mutation burden [6]. This hinders the ability to build reliable transcriptomics models.

A key characteristic of non-small cell lung carcinomas is that intra-tumor variability can be as large as or greater than inter-personal tumor variability [6], and consequently, this high local mutational diversity negatively affects the robustness of transcriptomic data. In contrast, imaging features are calculated through the entire lesion, generating complex patterns that include features from different tissue types within the tumor’s microenvironment, such as hypoxic, oxygenated, and necrotic tissue. Therefore, the total mutational burden could potentially be captured by integrating both transcriptomics (cell-specific data) and radiomics (tumor patterns) into a single radiotranscriptomic signature. In particular, this complex signature could alleviate the limitations of single-source data by synthesizing a holistic representation that merges markers associated with biological pathways (transcriptomics) and tumor heterogeneity (imaging features) [7, 8].

Consequently, methods that capture the diverse tumor microenvironment can have a positive impact by accurately assessing high-level clinical outcomes, such as the therapy response and survival analysis of cancer patients. This might be a key advancement for personalizing treatment since it improves the probability of a successful outcome, helps identify patients that may require aggressive treatment, reduces healthcare costs, aids in preventing the use of ineffective therapies that may result in unwanted negative effects, and enhances the likelihood of patient survival with a better quality of life.

In the field of oncology, radiotranscriptomics has been used in a few studies to evaluate the survival of IDH1 wild-type glioblastoma patients [9], estimate the survival (progression-free and overall) of lung cancer patients [10] based on a nomogram analysis, assess the complementary nature of radiotranscriptomic markers of NSCLC [11], and predict the molecular and histological subtypes of NSCLC [8]. Overall, while the current literature provides important insights into the NSCLC response to different types of therapy, there are still several issues that need to be addressed, such as multi-omics integration, to improve the precision and generalizability of ML models.

In this study, multiple multi-omics analyses were employed to assess survival and the adjuvant chemotherapy response of NSCLC patients. The aforementioned analyses incorporate selected features from pretrained deep models, radiomics, transcriptomics, and clinical data into a robust, unified feature space. This is performed by a traditional early-fusion multi-omic integration and through a novel radiotranscriptomic score. The use of domain-independent machine learning (ML) algorithms to identify relevant biomarkers and multi-omic integration to predict high-level clinical outcomes has the potential to enhance prediction performance and stability.

## Results

The computational pipelines were executed on a processing infrastructure featuring a 32-thread AMD Ryzen processor with 64 gigabytes of memory and an Nvidia RTX 3090 graphics card.

The pixel-based region of interest (ROI) in the CT scan was utilized to obtain a deep feature vector on a slice-by-slice basis. This resulted in multiple feature vectors per patient. A max-pooling method was used on these vectors to calculate the volume-based features for each patient. Additionally, PyRadiomics [12] was used to extract the radiomics based on the volume of interest. Zero-variance features were discarded. To substantially limit the feature space and identify the most significant components for each clinical task in each distinct view (radiomics, deep features, and transcriptomics), the analysis of variance and logistic regression with L1 penalty were applied sequentially for feature selection. A synthetic oversampling technique (SMOTE) was applied individually to the selected feature vector of each view to mitigate the effect of class imbalance in the examined patient cohorts. The proposed fusion technique for multi-omics analysis includes the concatenation of all views into a single feature space or the computation of a multi-omics score prior to classification. Several machine learning classifiers, including k-NN, decision trees, Gaussian processes, and SVMs with multiple kernels such as sigmoid, linear, polynomial, and radial-basis functions, were employed interchangeably to assess the therapy response. Additionally, methods such as the Cox, tree-based, and SVM-based models were employed to perform the survival analysis. To this end, the classification tasks were performed with scikit-learn [13] and the survival analysis with the scikit-survival [14] library.

All of the experiments were based on the same experimental protocol but with different variables and data handling methods. In particular, the therapy response analyses led to 100,800 experiments (experimental variables: 1 multi-omics and 3 single-source by 18 deep models by 7 classifiers by 2 fusion strategies by 100 iterations), and the survival analysis with multiple classifiers yielded 142,000 experiments (experimental variables: 1 multi-omics and 3 single-source by 18 deep models by 10 classifiers by 2 fusion strategies by 100 iterations). In each iteration, the same experimental parameters and patient splits were used across all single-source and multi-omics models. Therefore, the overall results are directly comparable across the experiments. Additionally, among the iterations, the patients were shuffled randomly prior to applying k-fold cross-validation.

### Adjuvant therapy response

**Fig. 1:**
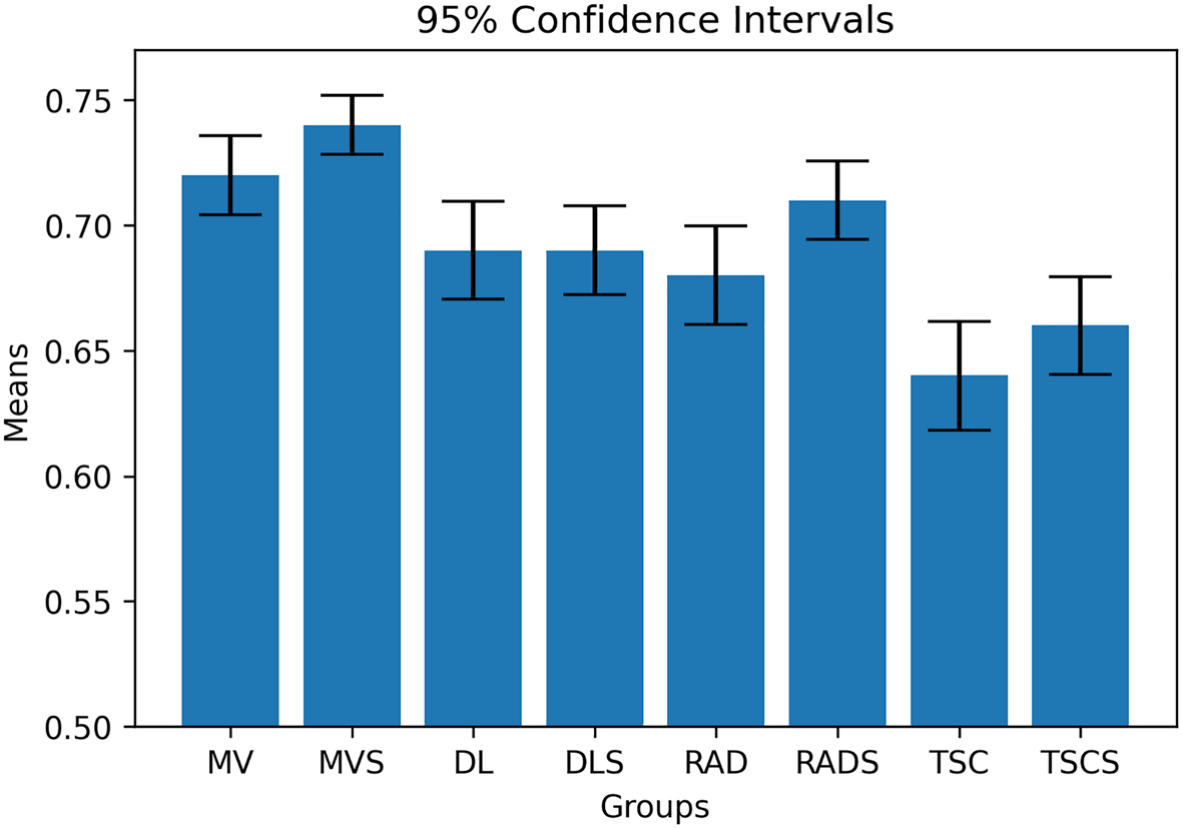
Performance comparison of therapy response models with 95% confidence intervals. *MV* multi-view, *MVS* multi-view score, *TSC* transcriptomics, *TSCS* transcriptomic score, *RAD* radiomics, *RADS* radiomic score, *DL* deep learning, *DLS* deep learning score

**Fig. 2:**
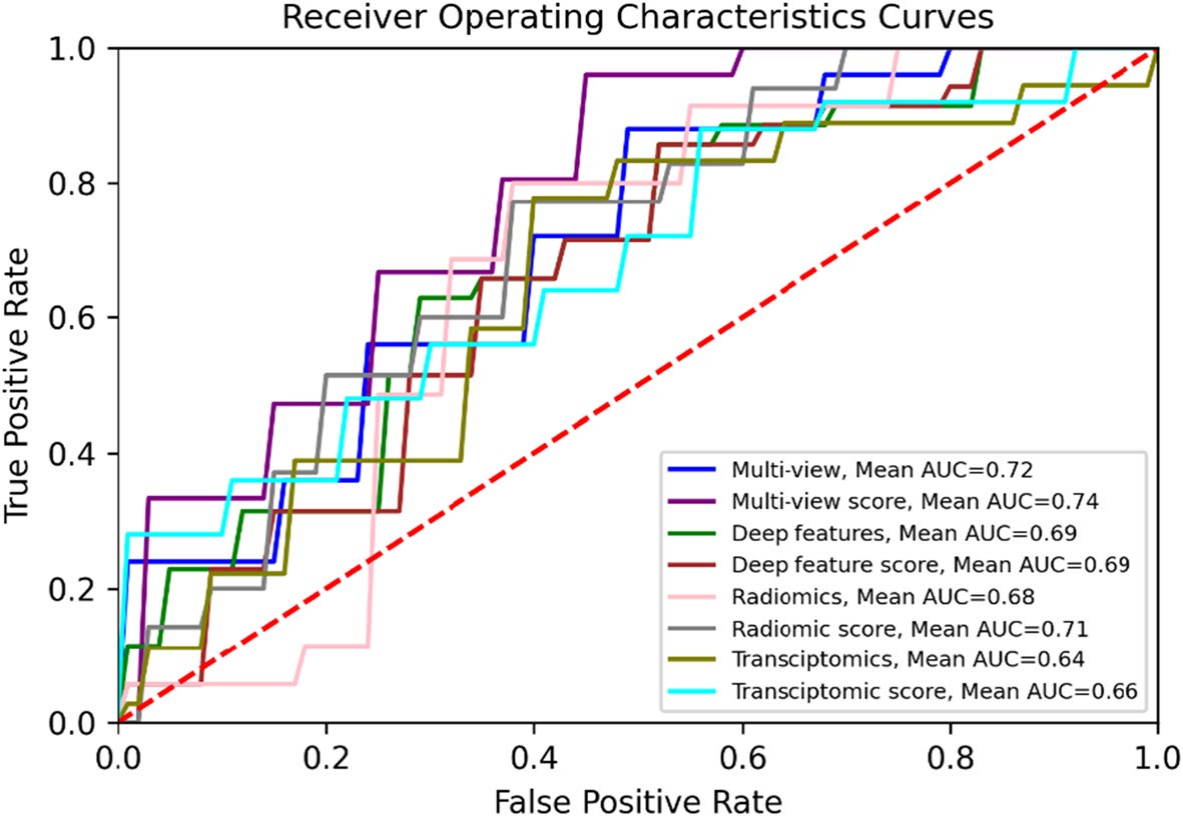
The receiver operating characteristic curves for the sigmoid SVM models are based on multi-view, single-source, and score-based analyses. *SVM* support vector machine, *AUC* area under curve

The performance of multi-view analysis in terms of AUC on the unseen testing set improved by 0.03–0.10 (Table 1) compared to the best single-source model, as seen in Figs. 1 and 2. Additionally, the score analysis based on the multi-view features achieved the lowest prediction variability, offering a more stable model compared to the corresponding single-source models. Deep features extracted from the NasNet architecture achieved the highest AUC score. Overall, the multi-omic score-based models outperformed the feature concatenation-based models both in terms of performance and prediction bias.

**Table 1:** Performance analysis of the SVM-based multi-view and single-source pipelines for adjuvant treatment response

	AUC	SN	SPC
Multi-view	0.72 ± 0.08	0.58 ± 0.2	0.61 ± 0.2
Multi-view score	0.74 ± 0.06	0.65 ± 0.08	0.62 ± 0.1
Deep features	0.69 ± 0.1	0.29 ± 0.2	0.84 ± 0.15
Deep feature score	0.69 ± 0.09	0.71 ± 0.19	0.57 ± 0.2
Radiomics	0.68 ± 0.1	0.72 ± 0.2	0.51 ± 0.15
Radiomic score	0.71 ± 0.08	0.70 ± 0.15	0.56 ± 0.18
Transcriptomics	0.64 ± 0.11	0.69 ± 0.2	0.38 ± 0.2
Transcriptomics score	0.66 ± 0.1	0.63 ± 0.15	0.55 ± 0.13

The following metrics represent the mean ± standard deviation of 100 iterations for each pipeline. *SVM* support vector machine, *AUC* area under curve, *SN* sensitivity, *SPC* specificity

After applying a zero-variance threshold, the 66th, 890th, and 906th deep activations were among the highest-ranked features. The best radiomics include elongation, gray-level co-occurrence matrix cluster shade, gray-level size zone matrix gray-level non-uniformity, gray-level co-occurrence matrix maximal correlation coefficient, gray-level size zone matrix entropy, and gray-level dependence matrix variance extracted from wavelet-decomposed images. The highest-ranked transcriptomic signature comprised OTUD3, SUCGL2, and RQCD1. In total, six deep features, six radiomics, and three transcriptomics were ranked highly for assessing the therapy response.

### Survival analysis

**Fig. 3:**
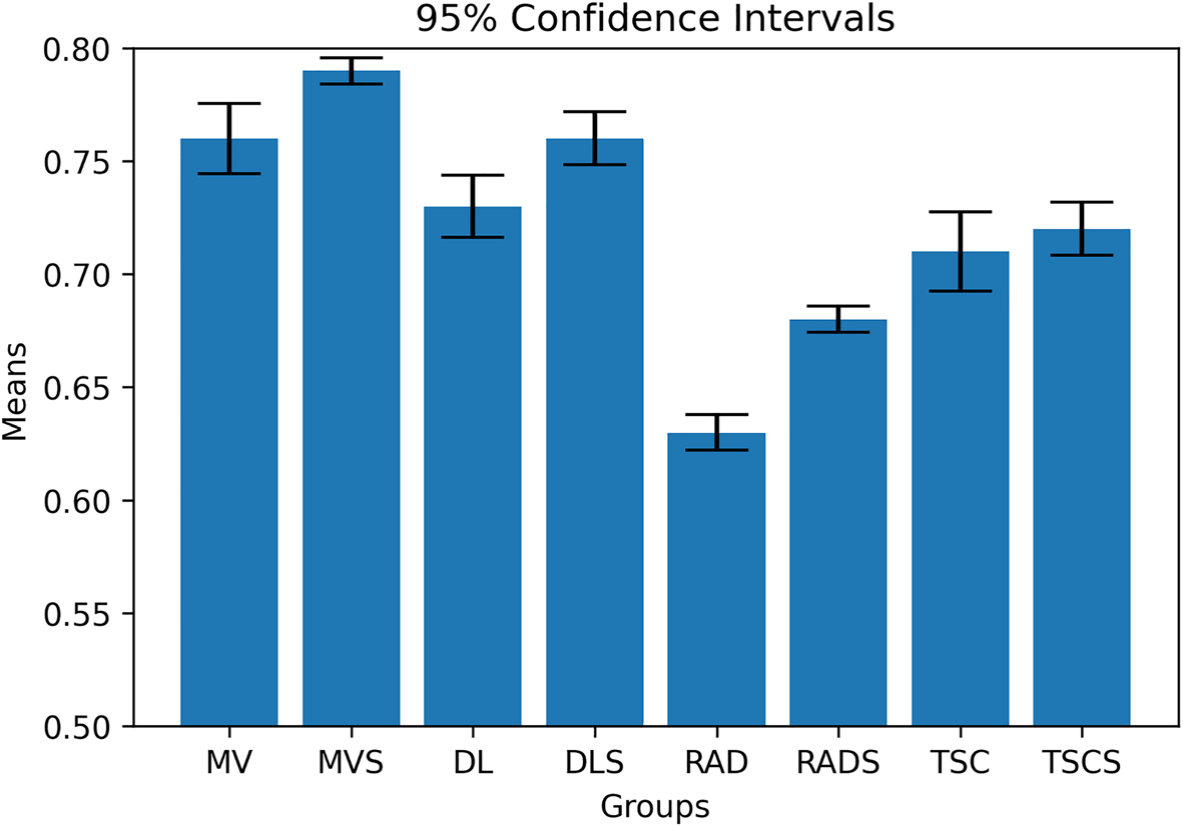
Performance comparison of survival analysis models with 95% confidence intervals. *MV* multi-view, *MVS* multi-view score, *TSC* transcriptomics,  *TSCS* transcriptomic score, *RAD* radiomics, *RADS* radiomic score, *DL* deep learning, *DLS* deep learning score

**Fig. 4:**
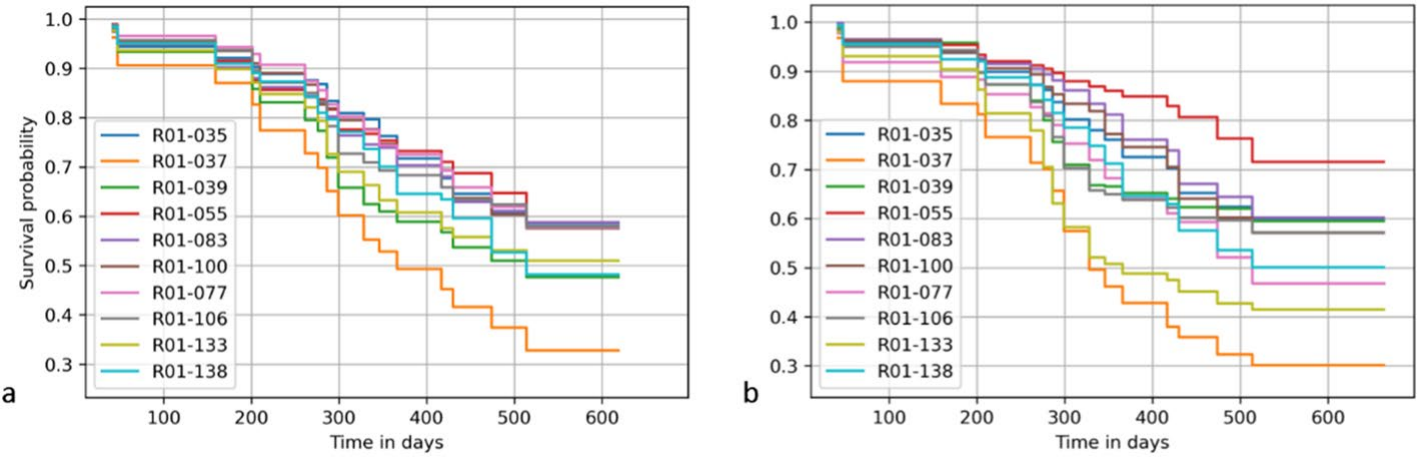
The predicted survival function of the multi-view analysis (**a**) and best single-source model (**b**). The per-patient probability of survival for high-risk patients (R01-037, R01-039 and R01-138) appear with a lower score in this figure compared to the low-risk patients (R01-035, R01-055 and R01-077). The deep feature score-based model (**b**) assigns high-risk patients with higher survival probability (R01-039, R01-106) and vice versa (R01-077)

The analyses with multi-source data performed better (improved C-index by 0.03–0.23) than the best models with a single data type and with overall lower variability (Table 2). The score-based analyses of both the multi-omics and single-source based models yielded better results with reduced prediction variability. In particular, the multi-omics scores outperformed the concatenation-based methods with the exception of some simpler models such as Cox, CoxPH, and survival tree, as shown in Table 2 and Fig. 3. The predicted survival functions based on the multi-omics for patients from the unseen testing set are depicted in Fig. 4a. High-risk patients like R01-037, R01-039, and R01-138 are presented with a low survival probability. This is in contrast to low-risk patients such as R01-035, R01-055 and R01-077 which are shown to have a higher survival probability.

**Table 2:** Performance analysis of the proposed multi-view and single-source pipelines for survival analysis

	Cox	CoxPH	Extra trees	Survival tree	Random forest	SVM-based
Multi-view	0.63 ± 0.11	0.68 ± 0.15	0.64 ± 0.06	0.64 ± 0.09	0.61 ± 0.12	0.76 ± 0.08
Multi-view score	0.64 ± 0.11	0.67 ± 0.05	0.69 ± 0.11	0.67 ± 0.12	0.66 ± 0.08	0.79 ± 0.03
Deep features	0.61 ± 0.08	0.62 ± 0.10	0.66 ± 0.12	0.64 ± 0.04	0.60 ± 0.04	0.73 ± 0.07
Deep feature score	0.66 ± 0.03	0.66 ± 0.04	0.62 ± 0.07	0.58 ± 0.14	0.57 ± 0.06	0.76 ± 0.06
Radiomics	0.61 ± 0.04	0.63 ± 0.04	0.63 ± 0.08	0.60 ± 0.09	0.57 ± 0.14	0.56 ± 0.05
Radiomic score	0.65 ± 0.03	0.63 ± 0.08	0.66 ± 0.05	0.61 ± 0.11	0.58 ± 0.08	0.68 ± 0.03
Transcriptomics	0.71 ± 0.09	0.70 ± 0.06	0.67 ± 0.05	0.68 ± 0.08	0.64 ± 0.04	0.71 ± 0.15
Transcriptomics score	0.69 ± 0.08	0.72 ± 0.06	0.67 ± 0.05	0.68 ± 0.01	0.64 ± 0.05	0.72 ± 0.09

The following metrics represent the mean ± standard deviation of 100 iterations for each pipeline. *CoxPH* Cox proportional hazards, *SVM* support vector machine

ResNet was the pretrained model that gave the highest-performing deep descriptors for this endpoint, including the 53rd, 64th, and 432nd activations. The radiomic signature for this endpoint incorporates gray-level run-length or size-zone non-uniformity and kurtosis from the original, biorthogonal, and daubechies wavelet images. Additionally, some of the transcriptomic features that were identified include ATIC, USP7, PNPLA2, ZZZ3, PGRMC2, TRAK1, and ELP3. Finally, seven deep features, six radiomics, and seven transcriptomics were used in the survival analysis.

## Discussion

Since its inception, AI has been developed to mimic the adaptation and self-organization of living organisms or biological structures for finding novel solutions and making decisions based on a data-driven framework. It is only a natural next step for AI to combine multiple sources of data for medical applications, which mirrors the decision-making process of an oncologist.

The vast majority of the current research [15–18] is based on image analysis tasks such as radiomics and only a handful of studies leverage the multi-modal nature of medicine. Radiotranscriptomics integration was also performed with nomograms [11] or feature concatenation techniques [8] as opposed to the proposed multi-omics score. The current study addresses these remarks by proposing an integrative multi-modal score to improve model robustness, examining the impact of a variety of non-linear classifiers for OS and therapy response, and incorporating the best practices in ML analysis to achieve fairness in model evaluation.

In particular, the proposed model integrates imaging data processed by qualitative (deep features) and quantitative (radiomics) methods, key clinical parameters (ethnicity, tumor location, histology grade, etc.), and transcriptomic features (cell-level information). The synergy of this diverse feature space captured the high intra-tumor variability and predicted important clinical endpoints such as patient survival and therapy response in a personalized manner. As discussed in the introduction, single-source models could potentially carry biases related to the feature extraction method. By incorporating diverse data sources, including radiomics (which captures tumor heterogeneity), transcriptomics (which provides cellular-level information), and selected clinical data (such as weight, age, and smoking status to assess overall health status), it is probable to mitigate certain adverse effects associated with the feature extraction method. This is shown by the performance delta between the multi-view and the corresponding single-source models for both clinical endpoints.

### Adjuvant therapy response

**Fig. 5:**
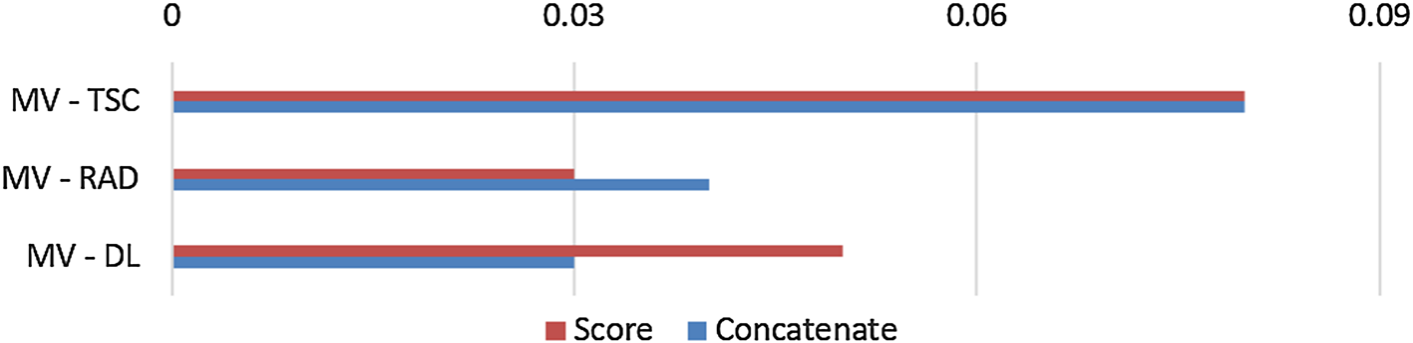
The differences between multi-view and single-source models in terms of AUC. *MV* multi-view, *TSC* transcriptomics, *RAD* radiomics, *DL* deep learning, *AUC* area under curve

For this task, the examined single-source models tend to be either highly sensitive with low specificity or highly specific with low sensitivity, depending on the data source, which can be interpreted as biased analyses. The combination of selected features from different sources led to a more robust model with an improved AUC and, most significantly, a better balance between sensitivity and specificity. Furthermore, Fig. 5 demonstrates that the differences in performance between multi-view analysis and any single-source model favor the former.

The highest-ranked features that were identified in this study have also been strongly associated with therapy response in other published studies with different experimental protocols and on different NSCLC patient cohorts. In particular, the feature cluster shade quantifies the asymmetry, pixel intensity variability in the region of interest, and cavitation [19], all of which have been previously correlated with pathological therapy response [20]. Zone entropy reveals the heterogeneity of the texture and has been found to be strongly correlated with histology subtypes [21]. A link between therapy response and the high value of dependence variance was identified in a recent study [22]. A correlation was found between elongation and local control of the tumor [23], which essentially means that the growth and spread of the tumor had been halted as a result of the treatment. The identified transcriptomics have also been featured in the current literature. In particular, OTUD3 has been linked to chemotherapy response [24], RQCD1 has been linked to tumor growth by uncontrolled activation [25], and SUCGL2 has been linked to tumor growth in cells with low glucose uptake [26].

### Survival analysis

**Fig. 6:**
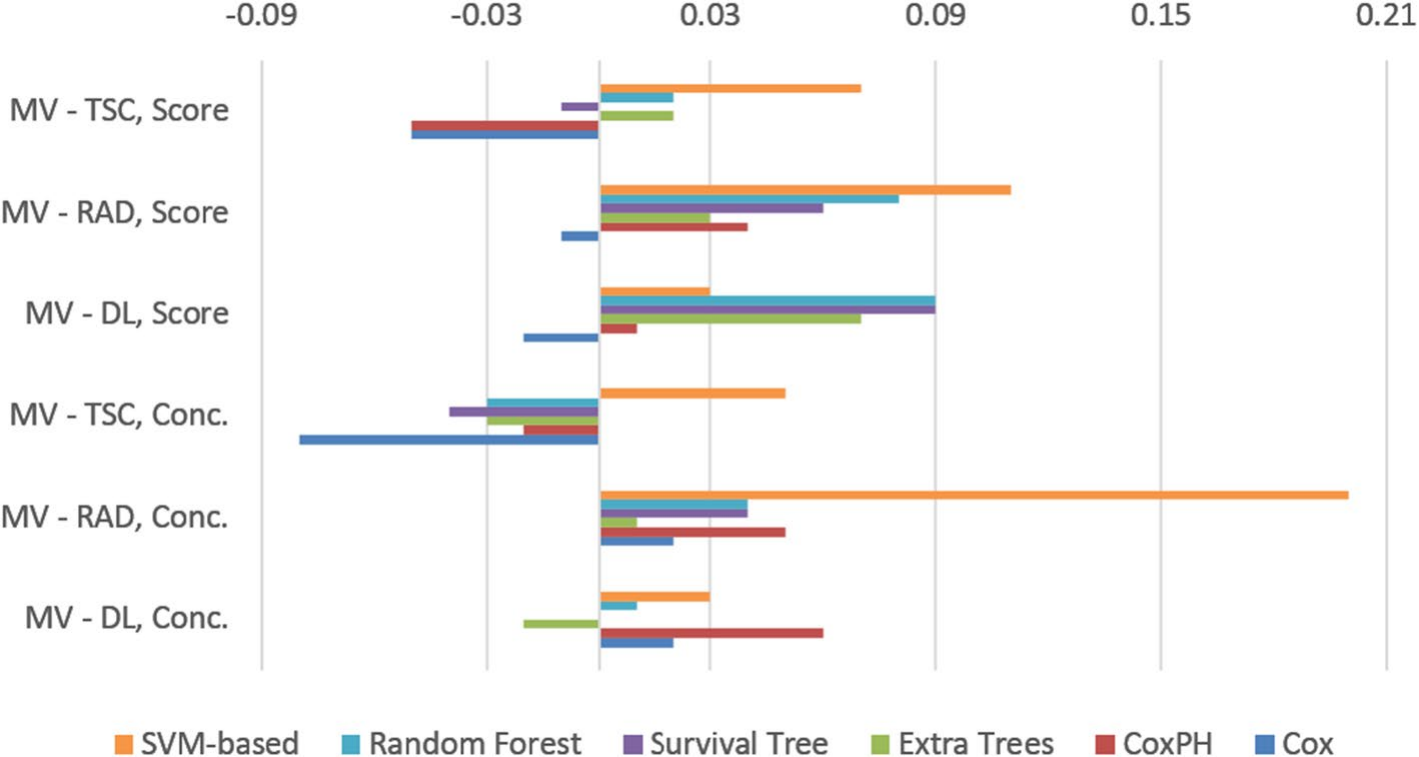
The differences between multi-view and single-source survival models in terms of concordance index. *MV* multi-view, *TSC* transcriptomics, *RAD* radiomics, *DL* deep learning

In terms of the concordance index, the SVM-based survival model performed best, with a C-index of 0.79 ± 0.03, as shown in Fig. 6. The improvements in C-index performance range from 0.03 to 0.20 in favor of the multi-omics SVM models compared to their single-source counterparts, as depicted in Fig. 6. Notably, the multi-omics models performed substantially better when paired with a classifier that can exploit complex relationships among features, such as the SVMs. This can be attributed to the high diversity of the multi-omics signature. Simpler classifiers performed better in a few single-source models, such as transcriptomics, but did not yield high performance overall.

Several radiomic features, including non-uniformity, have been strongly linked with survival probability in previous studies [27–31]. Kurtosis has been associated with overall and disease-free survival [32, 33]. The transcriptomics that have been previously reported for having an active biological role in NSCLC tumors and were identified as good predictors in this study include ZZZ3 which regulates biosynthetic activity via ribosome protein genes [34], PGRMC2 which has been linked to therapy resistance in adenocarcinomas and a lower patient survival rate [35], TRAK1 which has been associated with mitochondrial trafficking in cancer invasion cases [36] and has been identified as an emerging biomarker [37], and, finally, ELP3 which has been correlated with reduced cell growth in ALK-positive tumor cells [38].

### Limitations

The small number of patients with clinical data that corresponded to the examined high-level clinical outcomes that were available for this study limited the predictive power of the statistical methods used. Some secondary clinical variables were filled in with the median value (e.g., weight) or were randomly generated by combining multiple feature columns, such as the days survived for the alive patients. Additionally, some patients were rejected due to the lack of completeness in the data sources (imaging, transcriptomics, and other semantic data). This is a major limitation of this study and could be mitigated by utilizing a different approach, such as late fusion integration at the classifier level or other meta-estimator methods, at least for the therapy response analysis. Currently, transcriptomics is not routinely used data and is underutilized in clinical practice. In particular, it is more likely that centers in emerging economies or underdeveloped regions will have limited or no access to this type of data due to high costs and a lack of the required expertise in the field. The different data acquisition protocols in both imaging and transcriptomics could potentially harm the generalizability of the trained models since the proposed models were only trained with mostly homogeneous data from a single clinical site. Furthermore, transcriptomics is subjected to high intra-tumor variability. Therefore, a robust computational protocol is required. The semantic and clinical features might be subjected to inter-observer variability or other types of biases related to social epidemiology factors.

### Future extensions

To further validate this methodology, the modeling of multi-omics could be applied to other types of therapies, such as immunotherapy, and could model other types of tumors in different anatomical regions with varying genetic traits. In terms of multi-centric studies, CT examinations provide the most resilient imaging data, but incorporating data from scanners from different vendors should improve the generalization ability of the AI models. The same can be said for transcriptomics, although these types of data are highly dependent on the high-throughput computation protocol used, which might make data harmonization much more challenging. Lastly, incorporating a wider range of other semantic features could lead to a human-centric AI model, which has the potential to improve the interpretability and trustworthiness of this method.

## Conclusions

The proposed multi-omics analysis can potentially improve the prediction variability and accuracy of the two examined high-level clinical outcomes compared to the corresponding single-source models. Radiotranscriptomics, in conjunction with key clinical features, has the potential to capture a holistic representation of the tumor’s underlying biological mechanisms, as shown by the improved performance of OS and response to adjuvant therapy. A combination of these data sources has been shown to have a complementarity and synergetic effect, reducing the potential bias of single-source models and providing a highly discriminative signature across different clinically significant tasks.

## Materials and methods

### Patient cohort

NSCLC Radiogenomics [39] is a publicly available and unique dataset comprising imaging, genomics, transcriptomics, and clinical data that was created in order to promote the uncovering of the fundamental connection between transcriptomic/genomic and medical imaging and the development or assessment of predictive image biomarkers. As part of their care, patients underwent preoperative CT examinations at Stanford University Medical Center and Palo Alto Veterans Affairs Healthcare System. Varying scanners were used with an X-ray tube current of 124–699 mA (mean 220 mA) at 80–140 kVp (mean 120 kVp). Tumor samples were obtained from untreated patients during surgery. Within 30 min of excision, the removed tissue was frozen in a 3- to 5-mm-thick slice along the longest axis. Afterwards, it was recovered for RNA extraction. Molecular information such as gene expression microarrays, RNA sequencing, and mutational data on various oncogenes is also available for a subset of patients.

In particular, the examined dataset includes 211 CT (*P*_CT_) examinations accompanied by 142 pixel-based (*P*_ROI_) tumor annotations, RNA-seq data for 130 patients (*P*_TRANS_), clinical data (*P*_CL_) regarding the histology and molecular subtypes, treatment (*P*_T_), disease recurrence (*P*_DR_), survival status, and days (*P*_D_). For the patients with the survival status “alive”, the number of days was filled in randomly between ± 365 days of the maximum survival days in this dataset.

The multi-view analysis cohort comprised patients with a complete set of the abovementioned imaging, transcriptomics, and clinical data for the survival analysis cohort *P*_OS_ = *P*_CT_ ∩ *P*_ROI_ ∩ *P*_TRANS_ ∩ *P*_CL_ ∩ *P*_D_ and treatment response *P*_TR_ = *P*_CT_ ∩ *P*_ROI_ ∩ *P*_TRANS_ ∩ *P*_CL_ ∩ *P*_T_. The ∩ symbol denotes the intersection of sets, in this case the subset of patients among sets that have available data from multiple sources.

**Fig. 7:**
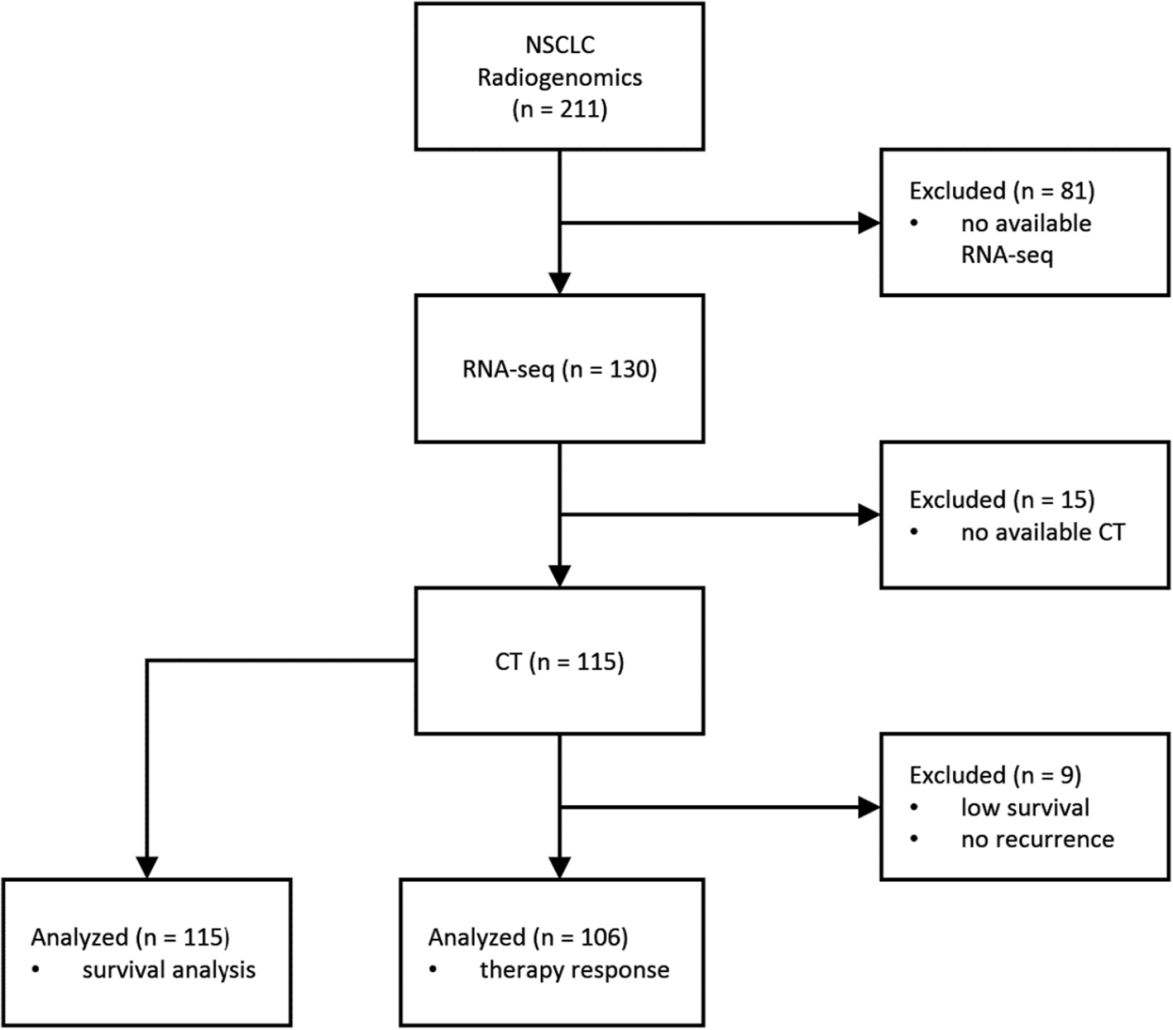
The CONSORT diagram of the study. *NSCLC* non-small cell lung cancer, *CT* computed tomography

The overall survival (OS) rate was calculated by taking the median of all the patients who were still alive after treatment and dividing them into two groups: those with a high survival rate (*L*_OS-H_ = *P*_OS_ ∩ [*P*_i_ > MEDIAN(*P*_D_)] = 92) and those with a low survival rate (*L*_OS-L_ = *P*_OS_ ∩ [*P*_i_ <  = MEDIAN(*P*_D_)] = 23). Since there are no follow-up imaging data available for the studied dataset, the treatment response was assessed by the survival rate in conjunction with disease recurrence (*L*_TR-POS_ = *P*_TR_ ∩ [(*P*_DR_ =  = False) AND *L*_OS-H_] = 68, *L*_TR-NEG_ = *P*_TR_ ∩ [(P_DR_ =  = True) AND *P*_Os_] = 38. In Fig. 7, a detailed CONSORT diagram depicts the patient selection process and criteria.

**Algorithm 1:**
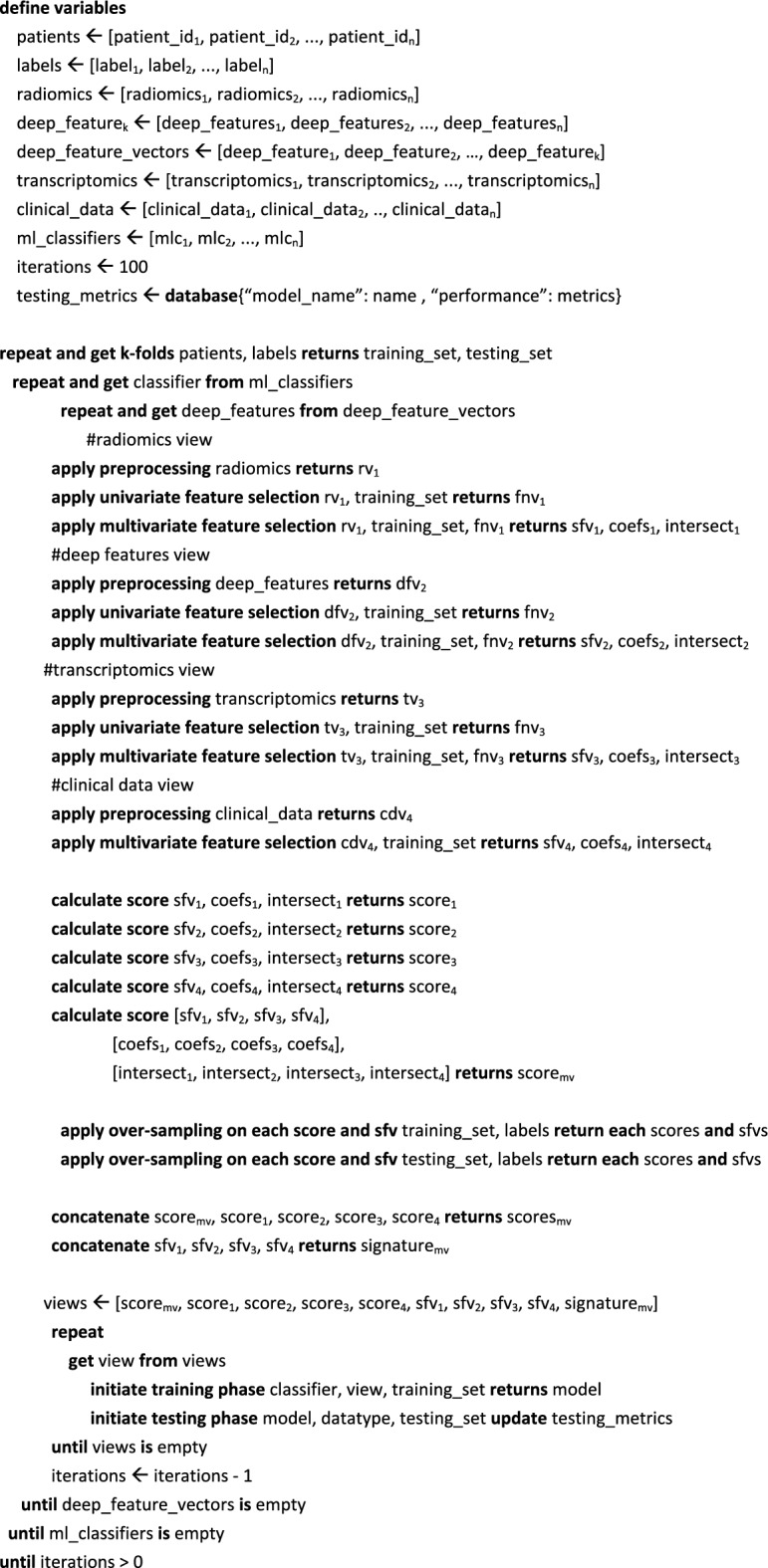
A simplified snippet of pseudo-code for the multi-view pipeline and single-source models

### Multi-view analysis

This study investigates the impact of merging multiple views, such as deep features, radiomics, transcriptomics, and key clinical data. Each view is a separate analysis for each feature type, presented as vectors in Eq. 1. Two types of feature integration were employed in this study: (a) a coefficient-based multi-omics score and (b) a signature of concatenated features of different views, as shown in Algorithm 1. The Lasso regression-based coefficient vector, shown in Eq. 2, of the selected features was used to generate a score from each view (Eq. 3). In particular, the multi-omics score is a mathematical algorithm that involves the selected features from multiple views and their coefficients, which are calculated using the Lasso method. This approach represents a higher level of integration when compared to the concatenated signature, which only involves the raw feature values from multiple views.

**Fig. 8:**
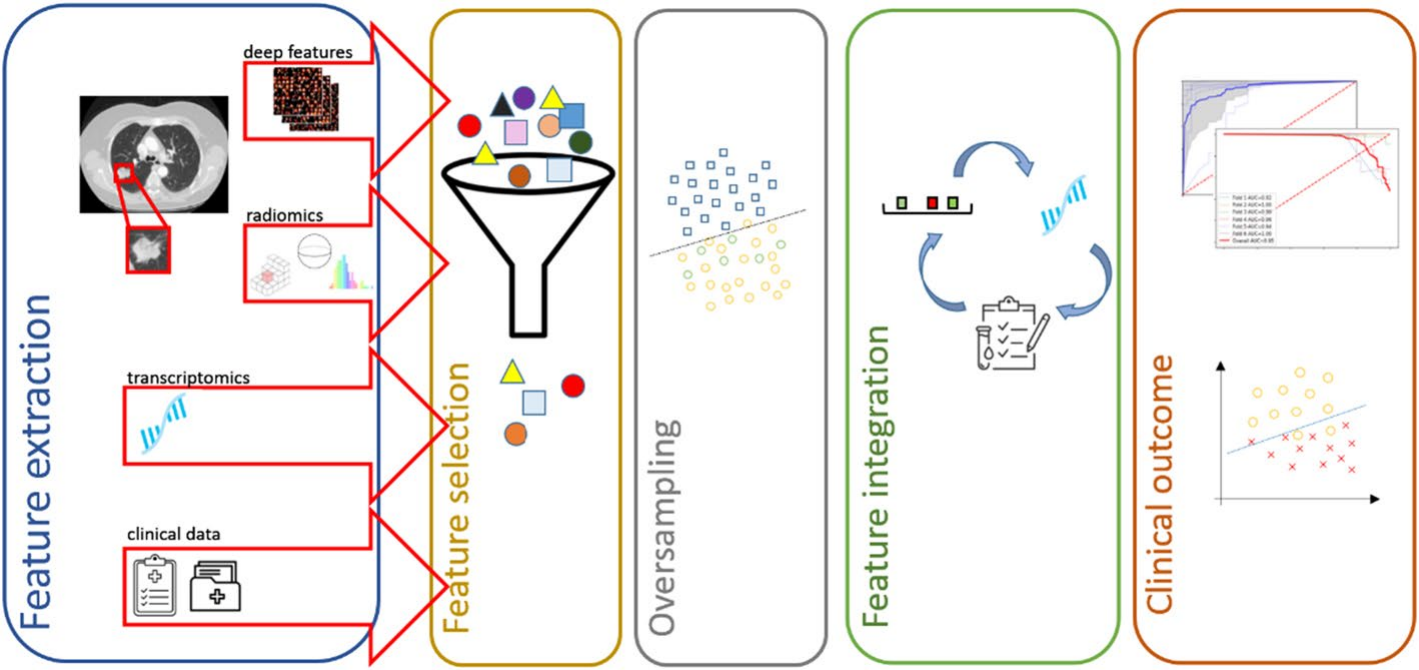
The proposed multi-view analysis for assessing high-level clinical outcomes. This pipeline includes feature extraction from multiple sources, followed by feature selection to identify the most relevant features to the specific clinical endpoint. SMOTE was applied to balance the examined distributions on the training set. Feature integration provides unified, compact representations of patient data for machine learning classification, assessing high-level clinical outcomes. *SMOTE* synthetic minority oversampling technique

In an alternative pipeline, early integration was implemented by concatenating (Eq. 4) the selected features from each view into a single feature vector prior to the survival analysis and therapy response classification, as presented in Fig. 8. Different regression-based scores [40] were calculated by using the corresponding coefficients of the selected transcriptomics, radiomics, deep feature vectors, and multi-omics (Eq. 5). In particular, the multi-omics signature is a vector that comprises the multi-omics score and each of the single-source scores (radiomics, deep features, clinical, and transcriptomics), as shown in Eq. 5. This is an extension of the methodology presented in our previous work [8], where only two sources of data were used for different endpoints. The source code can be publicly accessed online (https://github.com/trivizakis/multi-omics-nsclc/).

1 $${\text{features}}= \left[{f}_{1}, {f}_{2}, \dots , {f}_{n}\right]$$

2 $${\text{coefficients}}= \left[{c}_{1}, {c}_{2}, \dots , {c}_{n}\right]$$

3 $${{\text{Score}}}_{i}=\sum_{i=1}^{n}{{\text{intercept}}}_{i}+{{\text{features}}}_{i} \times {{\text{coefficients}}}_{i}$$

4

5 $${{\text{Score}}}_{{\text{multi}}-{\text{omics}}}= \left[\left(\sum_{i=1}^{n}{{\text{Score}}}_{i}\right), {{\text{Score}}}_{1}, {{\text{Score}}}_{2} , .., {{\text{Score}}}_{n}\right]$$

In the above equations, the square bracket represents a vector of multiple quantities, the multiplication of two quantities is denoted by $\times$, the addition of two values is denoted by + , concatenation of sequences is denoted by the  symbol, and summation is denoted by Σ.

### Imaging features

Two types of imaging features were extracted from the volume of interest of the CT examination: (a) deep features and (b) traditional radiomics. An “off-the-shelf” transfer learning (TL) technique was selected due to the low number of patients in the examined cohorts, limiting de novo network adaptation. A set of 18 pretrained deep models with ImageNet weights, featuring diverse architectures and internal representations, was employed. These include architectures such as Inception [41], Xception [42], DenseNet [43], ResNet [44], MobileNet [45], NasNet [46], and VGG [47], as well as their derivatives accessed from the online repository of Keras [48]. Only the convolutional layers of the pretrained models were transferred to the new deep model, allowing the extraction of feature maps from the low-level learned kernels. Deep feature extraction was employed on a slice-by-slice basis, and maximum pooling was performed on a patient-by-patient basis, yielding a latent space representation of the volume of interest. In particular, each slice was cropped around the normalized region of interest and then padded with zeros, resulting in a pixel array of 150 by 150 pixels. Prior to the analysis, deep features with zero-variance were discarded, substantially shrinking the extracted feature vector.

The radiomics consisted of 2996 features retrieved with a fixed bin size from the volume of interest of the CT scan. In addition to the 18 first-order features of skewness, energy, entropy, kurtosis, and other statistical features, 14 shape features, such as elongation, flatness, sphericity, 3D and 2D diameter, mesh, surface, and voxel volume, were computed. Seventy-five texture-matrix-based features were extracted, including autocorrelation, cluster prominence, contrast, gray-level covariance (GLCM), dependence (GLDM), run length (GLRLM), size zone (GLSZM), and neighborhood gray-tone difference (NGTDM). Version 2.2.0 of the PyRadiomics library [12] was used for extracting those features. Prior to extraction, isotropic resampling was applied to the examinations to ensure uniform spacing among all the imaging data. Six filtering techniques, such as exponential, gradient, Laplacian of Gaussian, square, square root, and wavelet filtering (twenty-two), enhanced the radiomics extraction by augmenting the extracted feature vector. The mother wavelets include daubechies, symlets, coiflets, biorthogonal, and reverse biorthogonal with two levels of decomposition [49]. Before the analysis, both deep features and radiomics were standardized on a feature basis.

### Transcriptomics

The RNA-seq data were retrieved from the NCBI GEO database [50] by comparing the patients’ pseudonyms with the NSCLC Radiogenomics imaging database. The pre-processing of the raw RNA-seq data resulted in the removal of genes that were missing values, resulting in a transcriptomic signature of 5268 genes for 130 patients.

### Clinical data

To avoid further reducing the already size-limited patient cohort, only complete features such as age, gender, weight, ethnicity, tumor location, number of pack years, smoking status, and histological grade were considered for the analyses. Additionally, for the survival analysis only, the categorical variable for the therapy type was included in the clinical feature view.

### SMOTE

Imbalanced distributions are a prevalent issue in data analysis and especially in medicine, since “normal” cases greatly surpass “odd” ones. It is quite common in machine learning tasks that these imbalances can have an adverse effect on the minority class, leading to less sensitive classifiers. The synthetic minority oversampling technique (SMOTE) [51] utilizes k-nearest neighbors to create samples by generating synthetic instances between two real ones near the boundaries of the hyperplane. This oversampling method was used in both the training and testing sets, but it was performed independently for each set to prevent data leakages.

### Feature selection

A combination of univariate and multivariate methods was utilized to select the most relevant features for each of the examined clinical outcomes. Initially, a zero-variance threshold was applied to the deep features and radiomics. Separately, for transcriptomics and imaging features, the analysis of variance (ANOVA) was utilized to determine the most significant features on a feature basis, aiming to reduce the predictors and avoid overfitting. Furthermore, a linear regression with an L1 penalty, also known as least absolute shrinkage and selection operator (Lasso) regression, was employed to decrease the length of each view by minimizing the coefficients, resulting in a compact representation.

### Data stratification

Four-fold cross-validation on a patient basis was employed to split the patient cohort into four pairs for training and testing, with class balances preserved across sets. The training set was solely utilized to perform feature selection, create synthetic instances of samples from the minority class, and fit the models. The testing set was only used for evaluating the model and remained unseen across the pipeline. This strategy was employed to improve the reliability of the followed experimental protocol, fairly assess the performance, and therefore avoid overfitting. Additionally, each pipeline was performed 100 times on different dataset splits, and consequently, the following results are presented in the form of a mean ± standard deviation (minimum–maximum) to better assess the performance of the proposed methodology. For the survival analysis, the cohort consisted of 80% of patients with high and 20% of patients with low survival probabilities. For the therapy response, the class distribution was 64% responders and 36% non-responders.

### Assessing clinical endpoints

Several machine learning classifiers were used for assessing the therapy response, including: (a) k-NN, (b) decision tree, (c) RBF-GPC, and SVMs with kernels such as (d) radial-basis function, (e) linear, (f) polynomial, and (g) sigmoid. For survival analysis, ten methods were employed based on (a) Cox, (b) Cox proportional hazards (CoxPH), (c) survival tree, (d) random forest, (e) extra trees, and also several SVM implementations of the scikit-survival [14], such as (f) linear kernel, (g) minimal Lipschitz smoothness strategy, (h) hinge loss, (i) fast survival, and (j) fast kernel SVM.

## Data Availability

The examined computed tomography and transcriptomic dataset titled “NSCLC Radiogenomics” is available online as an open-access repository via the following link: https://wiki.cancerimagingarchive.net/display/Public/NSCLC+Radiogenomics (accessed on 31 March 2019). The source code of the proposed analysis can be accessed online (https://github.com/trivizakis/multi-omics-nsclc/).

## Abbreviations

AI: Artificial intelligence
ML: Machine learning
DL: Deep learning
NSCLC: Non-small cell lung cancer
WHO: World Health Organization
CT: Computed tomography
RNA-seq: Ribonucleic acid sequencing
OS: Overall survival
PFS: Progression-free survival
NCBI GEO: National Center for Biotechnology Information gene expression omnibus
ANOVA: Analysis of variance
SMOTE: Synthetic minority oversampling technique
k-NN: K-nearest neighbors
RBF: Radial basis function
GPC: Gaussian process classifier
CoxPH: Cox proportional hazards
SVM: Support vector machine
AUC: Area under curve
SN: Sensitivity
SPC: Specificity
C-index: Concordance index
